# Supplementary material for: Active power filter control strategy based on repetitive quasi-proportional resonant control with linear active disturbance rejection control
Source: PLoS One. 2025 Feb 20;20(2):e0318558. doi: 10.1371/journal.pone.0318558 (PMC11841888; doi:10.1371/journal.pone.0318558)
Supplement: Attachment — Submitted filename: plos_latex_template.pdf [file pone.0318558.s001.pdf]

Active power filter control strategy based on repetitive quasi-proportional resonant control with linear active disturbance rejection control

Yifei Gao[1], Liancheng Zhu[1]*, Xiaoyang Wang[1], Hongshi Wei[2], Xiaoguo Lv[2]

1 School of Electrical Engineering, Liaoning University of Technology, Jinzhou, Liaoning Province, China
2 Research and Development Department, Liaoning Rongxin Xingye Electric Power Technology Company Limited, Anshan, Liaoning Province, China

* dq_lczhu@lnut.edu.cn (LZ)

Abstract

Active power filter (APF) is a new type of harmonic mitigation device, and its harmonic mitigation capability mainly depends on the control strategies of current inner loop and voltage outer loop. Traditional proportional integral (PI) control methods cannot track harmonic currents without steady-state error, thereby it leads to the poor tracking accuracy of the harmonic current and is difficult to achieve high-performance harmonic compensation. Thus, in this paper, a repetitive quasi-proportional resonant (QPR) with linear active disturbance rejection control (LADRC) is proposed. Firstly, QPR control is introduced to replace PI control. QPR control can eliminate steady-state error and realize no static error tracking of harmonic current. Meanwhile, QPR control can coordinate control of various frequency components and enhance APF 's ability to suppress specific harmonics. Then, the introduction of repetitive control optimizes the QPR control, so that the dynamic performance of the system does not change while further improving the tracking accuracy of the APF system for harmonic current. Finally, LADRC control is used to observe and compensate for the coupled parts of the system, thereby, it can achieve high-performance decoupling without adding additional sensors. In addition, in order to ensure the stability of the DC-side voltage and enhance its dynamic performance, this paper also designs a voltage outer loop controlled by QPR. The proposed method is verified by real-time digital simulation system (RTDS). The simulation results show that compared with the double closed-loop PI control, the repetitive quasi-proportional resonant with linear active disturbance rejection control (repetitive QPR-LADRC) and QPR double closed-loop control proposed in this paper can not only increase the harmonic current tracking accuracy of APF system, but also improve the dynamic performance of APF system and significantly enhance the harmonic suppression ability of APF.

Introduction

With the rapid development of modern social science and technology, a large number of nonlinear power electronic devices have been applied,and it results in a large number of harmonics and reactive power being injected into the public power grid. Excessive harmonic currents can cause damage to power electronic devices and even lead to safety accidents,then it will seriously affect the safe and stable operation of the public

grid [1, 2]. Currently, the mainstream harmonic suppression devices are passive power filter (PF) and active power filter. The structure of the passive power filter is simple, and its manufacturing cost is relatively lower. However, its harmonic suppression performance is limited by component parameters, and only 80% of harmonics can be filtered out at most [3]. Active power filter has a high compensation accuracy and excellent real-time performance. In theory, APF can completely compensate the harmonics of the power grid. And now, it has become the most important means of grid harmonic suppression [4, 5]. The introduction of three-level technology has further expanded the range of applications for APF [6].

The working principle of the active power filter is generating a compensation current with equal amplitude and opposite phase according to the detected command current, and then the compensation current is injected into the power grid for compensation. This requires that APF can track harmonic current in real-time, and the harmonic current tracking performance of APF mainly depends on the control strategy of current inner loop [7, 8]. At present, the control method often used in active power filter is to convert the AC signal into a DC signal in the synchronous rotating coordinate system through the Clark transform, and then the PI controller is used for tracking control. However, this control method has static tracking error and current coupling between the $d-$axis and $q-$axis, which ultimately leads to low harmonic current tracking accuracy [9]. Therefore, some scholars have developed a composite control strategy by combining repetitive control with PI control. This method takes into account the advantages of repetitive control and PI control, so it has high steady-state accuracy and fast dynamic performance. However, APF system still has the problem of incomplete decoupling [10]. The quasi-proportional resonance control based on the inner membrane principle has a high gain at its resonance point, so it can achieve zero steady-state error control. In [11], a double closed-loop control strategy based on PI and quasi-proportional resonance is proposed. This method realizes no static error tracking of the command current and improves the waveform quality of the output current. Based on the study of quasi-proportional resonant control, a multi-quasi proportional resonant (MQPR) control is proposed. The multi-quasi proportional resonant control can automatically change the resonant gain according to the inductive load and capacitive load, therefore it improves the compensation performance of APF [12]. In [13], a compound control combining fast repetitive control and PR control is proposed. This method can improve the compensation accuracy while maintaining the dynamic performance of proportional resonant (PR), and it effectively reduces the tracking error of the system. Ref. [14] improves the QPR controller and proposes an adaptive quasi-proportional phase compensation resonant control strategy. This control strategy not only enhances the system's robustness to grid frequency variations, but also strengthen the system's ability to suppress specific harmonics. In summary, the use of QPR control or related composite control strategies can effectively improve the tracking performance of the system, but there is a current coupling problem in the above control methods, and if the decoupling control cannot be achieved, the $d-$axis and $q-$axis will interfere with each other and affect the control performance [15].

Currently, the most common decoupling control strategy is feedforward decoupling control,which can achieve complete decoupling of the $d-$axis and $q-$axis. But the use of feedforward decoupling control will increase the cost of sensors [16]. In [17], a decoupling control strategy based on generalized inverse system method is proposed. This strategy can configure the system poles by adjusting the generalized inverse parameters and have a good decoupling control effect, but it is difficult to design the controller in practical application. Active disturbance rejection control (ADRC) is a control method that does not rely on precise system models, and it has strong anti-disturbance capabilities in the face of changes in system model parameters or unknown disturbances [18, 19]. Due to

the fact that there are many ADRC parameters and no direct relationship between them, a linear active disturbance rejection control is usually used to replace it in practical application. LADRC inherits the advantages of ADRC and reduces the number of parameters that need to be tuned by linearizing them [20]. Ref. [21] proposed a repetitive and LADRC control, where LADRC compensates for the current coupling part as an internal disturbance and decouples it to improve the harmonic current tracking accuracy of the APF system. By connecting deadbeat control with repetitive and LADRC in series, deadbeat repetitive LADRC control is formed. This control strategy can suppress odd harmonics, shorten the cycle of repetitive control and further improve the steady-state current tracking accuracy of APF system [22]. In [23], PI control is used to replace the linear state error feedback (LSEF) control, then a PI and linear extended state observer (LESO) decoupling control method is formed. Due to the use of fourth-order LESO, the control method can achieve high-performance decoupling control of the system, but there are many parameters to be tuned.

Combining the advantages of the above control strategies, this paper proposes a dual closed-loop control strategy suitable for neutral-point-clamped (NPC) type active power filter. The inner current loop adopts repetitive quasi-proportional resonant with linear active disturbance rejection control, where the former repetitive QPR control is used to eliminate steady-state error and improve harmonic current tracking accuracy, and the latter LADRC control is used to observe and compensate current coupling to achieve high-performance decoupling control. Compared with the PI control with feedforward decoupling control, the repetitive quasi-proportional resonant with linear active disturbance rejection control not only enhances the steady-state and dynamic performance of APF, but also reduces the sensor cost. The voltage outer loop adopts QPR control to improve the dynamic performance of the system under load changes and maintain the stability of the DC-side voltage. Finally, the effectiveness of the proposed double closed-loop control strategy is verified by real-time digital simulation system.

The second part of this paper establishes and analyzes the mathematical model of APF. In the third part, the current inner loop is analyzed and designed. In the fourth part, the voltage outer loop is analyzed and designed. In the fifth part, the feasibility of the proposed control strategy is verified by RTDS online simulation. Finally, the conclusion is given.

The establishment and analysis of APF mathematical model

Fig 1 is the topology of neutral-point-clamped three-level active power filter with LCL filter. In the diagram, u_{ga}, u_{gb} and u_{gc} represent the three-phase grid voltage; i_{fa}, i_{fb} and i_{fc} represent the harmonic current; L_{l1}, L_{l2} and C_{c1} are the inverter side filter inductance, the grid side filter inductance and the filter capacitance of the LCL filter respectively; R_{c1} is the damping resistance; i_{l1a}, i_{l1b} and i_{l1c} are the output current of the inverter; i_{l2a}, i_{l2b} and i_{l2c} are the output current of the inverter to the grid; C_1 and C_2 are the DC-side voltage dividing capacitor; i_n is the current flowing into the voltage dividing capacitor, and V_{dc} is the voltage of DC-side.

Fig 1. Topology of the three-level active power filter with LCL filetr.

Because the LCL filter has equal three-phase parameters and the three-phase power grid is symmetrical, the circuit in Fig 1 can be simplified. Considering that the filter resistance of the LCL filter is small, it is ignored for the convenience of analysis. Taking phase a as an example, the simplified APF single-phase equivalent circuit diagram is shown in Fig 2.

Fig 2. The single-phase equivalent circuit diagram of APF.

Where, u_{l1} represents the output voltage of the inverter.

In this structure, the LCL filter exhibits resonance phenomena, where the amplitude-frequency curve shows a resonance peak, and the phase-frequency curve shows a negative step of $-180°$, and it will lead to system instability. Therefore, this paper adds passive damping to suppress resonance in the system.

From Fig 2, according to Kirchhoff's voltage and current laws, the relationship between the output voltage u_{l1} of the inverter and the current i_{l2} flowing from the inverter to the grid side can be obtained [24].

$$G_{LCL}(s) = \frac{i_{l2}}{u_{l1}} = \frac{R_{c1}s + 1}{L_{l1}L_{l2}C_{c1}s^3 + (L_{l1} + L_{l2})R_{c1}s^2 + (L_{l1} + L_{l2})s} \tag{1}$$

From Eq (1), it can be seen that the active power filter with grid side current feedback control is a third-order system. Controlling a high-order system with a low-order controller is often difficult to achieve optimal control effects, and designing a high-order controller depends on the precise model of the controlled object [25, 26]. For this reason, the Padé approximation method is often used to approximate the first-order system model to the third-order system model, which helps to achieve a balance between control effect and design complexity. By using the Padé approximation method, Eq (1) can be reduced to Eq (2) [27, 28].

$$G_{LCL}(s) = \frac{i_{l2}}{u_{l1}} \approx \frac{1}{(L_{l1} + L_{l2})s} = \frac{1}{L_l s} = G_L(s) \tag{2}$$

From Eq (2), it can be obtained that after the Padé approximation method is used to reduce the order of the system model, the relative order of the system model becomes first-order [29, 30]. At this time, the LCL filter can be approximately equivalent to an L filter. Therefore, the mathematical model of APF can be simplified as follows [31]:

$$\begin{cases} L_l \dfrac{di_{l2d}}{dt} = u_{l1d} - u_{gd} + wL_l i_{l2q} \\ L_l \dfrac{di_{l2q}}{dt} = u_{l1q} - u_{gq} - wL_l i_{l2d} \end{cases} \tag{3}$$

As shown in Eq (3), there is a current coupling between the $d-$axis and $q-$axis in the APF mathematical model. If a decoupling control strategy is not adopted, the $d-$axis and $q-$axis will interact with each other, which will seriously affect the control performance.

Design and analysis of current inner loop

Design and analysis of QPR control

Due to the fact that traditional proportional resonant control cannot effectively respond to changes in grid frequency, so the QPR control is often used as an alternative in practical applications [32]. As a special case of PR control, QPR control adds a new zero point on the basis of PR control, so that the frequency characteristic curve near the resonant frequency changes from steep to smooth. This not only produces a higher

amplitude gain near the resonant frequency, but also enhances the anti-interference of the system [33].

The transfer function of QPR control is [34]:

$$G_{QPR}(s) = k_{p1} + \frac{2k_{r1}w_{c1}s}{s^2 + 2w_{c1}s + {w_{01}}^2} \tag{4}$$

where, k_{p1} is the proportional gain coefficient; k_{r1} is the resonance factor; w_{c1} is the sideband angular velocity of the controller; and w_{01} is the resonance frequency.

Substituting the resonant frequency into the $G_{QPR}(s)$, the gain at the resonant frequency can be obtained as:

$$A_{QPR}(jw_{01}) = k_{p1} + k_{r1} \tag{5}$$

From Eq 5, it can be seen that the gain of the QPR controller at the resonance frequency w_{01} is only related to k_{p1} and k_{r1}. Therefore, the resonance gain can be improved by adjusting the values of k_{p1} and k_{r1}, and then the static error can be eliminated to achieve no static error control.

Below, the impact of w_{c1} on the QPR controller is analyzed. If $k_{p1} = 20$, $k_{r1} = 100$, $w_{01} = 500$ rad/s, the Bode diagram of the QPR controller is shown in Fig 3.

Fig 3. Bode diagram of the QPR controller.

From Fig 3, it can be seen that the sideband angular velocity w_{c1} of the controller only affects the bandwidth and has no effect on the gain at the resonance. Furthermore, the larger w_{c1} is set, the larger the bandwidth at the resonance frequency will be.

When specific subharmonics need to be compensated, different w_{01} values need to be set for current tracking control, so multiple QPR controllers are generally required for control [35]. In practice, the higher the harmonic order, the less the harmonic current content, so it is usually only necessary to consider within 25 times [36]. This paper mainly enhances the ability of 5th, 7th and 11th harmonic suppression, so the QPR controller designed is shown in the Fig 4.

Fig 4. Schematic diagram of the QPR control.

Where, $w_{01} = 2\pi f$, f is the fundamental frequency, ${i_{l2}}^*$ represents the command current signal, and e_{l2} represents the tracking error.

Design and analysis of repetitive QPR control

The inner membrane theory is the core of the repetitive controller, and the essence of this theory is to implant the mathematical model of the dynamic characteristics of the command signal and the disturbance signal into the control loop to form a high-precision feedback control system [37]. Therefore, the use of repetitive control can greatly reduce the steady-state tracking error of AC system and improve the harmonic suppression capability of APF. The block diagram of the repetitive control structure based on the inner membrane in the z domain is shown in Fig 5.

Fig 5. The structure diagram of the repetitive control.

In Fig 5, the repetitive controller can be divided into two parts : the inner membrane structure and the compensator. $Q(z)$ and z^{-N} constitute the inner

membrane structure, $K_r z^k S(z)$ is the compensator, and the controlled object is $G_{l2}(s)$. Taking a too large $Q(z)$ often leads to the instability of the system [38]. Therefore, $Q(z)$ generally takes a constant less than 1, and this paper selects 0.95, N is the number of sampling points, and 400 is selected in this paper.

The design of the compensator is the most important part. K_r is the gain coefficient, which is usually set to 1; z^k is the leading link, and it is set to 4 in this paper. $S(z)$ is a second-order low-pass filter, and its main function is to suppress the high-frequency interference of the system and improve the stability of the system. Therefore, selecting an excessively high or low filter cutoff frequency is meaningless. An excessively high cutoff frequency may cause system instability, while a low cutoff frequency will reduce the APF system's ability to suppress high-frequency harmonics [39].

Considering that in practice filters are mainly used to compensate for harmonics up to the 25th order, the cutoff frequency w_n is set to 2.5 kHz in this paper. After the discrete approximation, the expression of the second-order low-pass filter is shown in Eq 6.

$$S(z) = \frac{0.2098z + 0.1443}{z^2 - 0.9753z + 0.3294} \tag{6}$$

Fig 6 shows the Bode diagram of the LCL filter $G_{LCL}(s)$ with feedback grid side current.

Fig 6. Bode diagram of the LCL filter.

From Fig 6, its frequency characteristics are similar to single inductor in the low frequency band (below 500). Therefore, this paper uses the differential link with inertia to offset, and the system transfer function after offset is shown in Eq 7.

$$\begin{aligned} G_{l2}(s) &= G_{LCL}(s) \times \frac{Ks}{\tau s + 1} \\ &= \frac{2.31 \times 10^{-8}s^2 + 0.0021s}{4.4 \times 10^{-16}s^4 + 6.82 \times 10^{-12}s^3 + 4.431 \times 10^{-7}s^2 + 0.0021s} \end{aligned} \tag{7}$$

According to Fig 5, the characteristic equation of the system with repetitive control can be obtained.

$$1 - Q(z)z^{-N} + G_{l2}(z)S(z)z^4 z^{-N} = 0 \tag{8}$$

According to the small gain theorem [40], it can be concluded that the system is stable when the characteristic roots of the system characteristic equation fall in the unit circle on the z plane (i.e., $|zi| < 1$). Then, Eq 8 is simplified to Eq 9.

$$\left|Q(z) - G_{l2}(z)S(z)z^4\right| < 1 \tag{9}$$

Finally, the stability of the repetitive controller is further verified by the Nyquist curve. The Nyquist curve of $G_{l2}(z)S(z)z^4$ is shown in Fig 7.

Fig 7. Nyquist curve of the $G_{l2}(z)S(z)z^4$.

Since $Q(z)$ is set to 0.95 in this article, a unit circle with a radius of 1 is drawn around the point (0.95, 0). From Fig 7, it can be seen that the Nyquist curve of the

$G_{l2}(z)S(z)z^4$ repetitive control system is contained within the unit circle, therefore,it satisfies the stability condition and deduces that the repetitive control system is stable.

By combining QPR control with repetitive control in parallel, a repetitive QPR composite control can be obtained. The composite control takes into account the advantages of QPR and repetitive control, which can quickly and stably track the AC component in the system without static error. Thereby, it can improve the harmonic suppression effect of APF system. The control block diagram of the repetitive QPR compound control is shown in Fig 8.

Fig 8. The structure diagram of repetitive QPR control.

Design and analysis of LADRC control

From Eq 2, it can be concluded that after the Padé approximation method is applied, the transfer function of the APF system with LCL filter is reduced to first-order, so a first-order LADRC controller can be used for control.

The first-order LADRC controller mainly consists of the tracking differentiator (TD), the linear state error feedback rate (LSEF) and the linear extended state observer. Among them, the main function of TD module is to smoothly transition the given input by extracting the dynamic characteristics of the input signal; LESO is responsible for observing the total disturbance of the system; LSEF is responsible for performing differential control of the input signal and output signal error. In order to improve the response speed of the system, the TD module is not used in this paper [41]. The control block diagram of the first-order LADRC without the TD module is shown in Fig 9 [42].

Fig 9. The structure diagram of first-order LADRC control.

In Fig 9, u^* is the reference signal that the controller needs to track, i_{l2} is the current output by APF to the grid side, w_q is the undetermined external disturbance, APF is the controlled object of the controller, and u_q is the voltage output by the controller.

According to the LADRC control block diagram shown in Fig 9, Eq 3 can be rewritten in the following form:

$$\begin{cases} \dfrac{di_{l2d}}{dt} = b_q u_{l1d} + f_{qd}(i_{l2d}, i_{l2q}, u_{gd}, w_q) \\ \dfrac{di_{l2q}}{dt} = b_q u_{l1q} + f_{qq}(i_{l2d}, i_{l2q}, u_{gq}, w_q) \end{cases} \tag{10}$$

Specifically, $b_q = 1/L_l$, f_{qd} and f_{qq} represent the sum of internal and external disturbances in the APF model, and it includes the current coupling disturbance between i_{l2d} and i_{l2q}, the model error disturbance after the order reduction using the Padé approximation method and the external uncertain disturbance w_q. Considering that the $d-q$ axis has the same LADRC control structure, for the sake of convenience, only the $d-$axis will be analyzed in the following.

Two state variables x_{q1} and x_{q2} are defined, where x_{q1} represents the current i_{l2} output by APF to the grid side; x_{q2} represents the total internal and external disturbances f_q of APF system. Suppose that f_q is differentiable, then, Eq 10 can be expressed as a state space equation.

$$\begin{cases} \dot{x_q} = Ax_q + Bu_i + E\dot{f_q} \\ y_q = Cx_q \end{cases} \tag{11}$$

where,

$$x_q = \begin{bmatrix} x_{q1} \\ x_{q2} \end{bmatrix}, A = \begin{bmatrix} 0 & 1 \\ 0 & 0 \end{bmatrix}, B = \begin{bmatrix} b_q \\ 0 \end{bmatrix}, C = \begin{bmatrix} 1 & 0 \end{bmatrix}, E = \begin{bmatrix} 0 \\ 1 \end{bmatrix}, u_i = u_{l1}, y_q = i_{l2}$$

Design and analysis of LESO

From Eq 11, the second-order linear extended state observer can be designed.

$$\dot{z_q} = (A_q - LC_q)z_q + B_q u_i + LCx_q \tag{12}$$

where

$$z_q = \begin{bmatrix} z_{q1} \\ z_{q2} \end{bmatrix}, A_q = A, B_q = B, C_q = C, L = \begin{bmatrix} \beta_1 \\ \beta_2 \end{bmatrix}$$

In Eq 12, z_{q1} is the sensing value of the current output from APF to the grid, which is used to track i_{l2}; while z_{q2} is the observation value of the total disturbance of APF system, which is used to track f_q. β_1 and β_2 represent the observer gain of LESO.

From Eq 12, the convergence rate of the linear extended state observer is controlled by the poles of $A_q - LC_q$. Therefore, according to the bandwidth parameter tuning method [43], as long as all the poles of $A_q - LC_q$ are configured at $-w_0$, the observation error of the system will converge to zero, and the fast non-overshoot tracking of the reference signal can be realized. The corresponding expected characteristic polynomial is shown in Eq 13.

$$\lambda(s) = |sI - (A_q - LC_q)| = (s + w_0)^2 \tag{13}$$

According to Eq 13, the elements in the output error feedback matrix L can be obtained as:

$$\begin{cases} \beta_1 = 2w_0 \\ \beta_2 = w_0^2 \end{cases} \tag{14}$$

where, w_0 is the LESO bandwidth.

Design and analysis of LSEF

LSEF can feed back the state observations of the system observed by LESO to the controller. Therefore, the total output of the controller can be expressed as:

$$u_q = \frac{u_{q0} - f_q}{b_q} \tag{15}$$

Among them, u_{q0} is the control input without disturbance compensation. When there is no observation error between the observed value z_{q2} and the actual value f_q, the system can be further simplified.

$$\dot{y_q} = u_{q0} + f_q - z_{q2} \approx u_{q0} \tag{16}$$

It can be seen from Eq 16 that the system has been corrected to an integral series structure, so only proportional controller can be designed to eliminate the static error.

$$u_{q0} = k_{pp}(v_q - z_{q1}) \tag{17}$$

Specifically, $v_q = u^*$ represents the signal to be tracked, k_{pp} is the gain of the controller, and the controller bandwidth $w_c = k_{pp}$ is often used.

By substituting Eq 17 into Eq 16, the closed-loop transfer function from the input reference signal to the final output can be obtained.

$$G_{vl}(s) = \frac{w_c}{s + w_c} \tag{18}$$

Where, w_c is the controller bandwidth and usually $(\frac{1}{10} - \frac{1}{3})$ times the value of w_0. It can be seen from Eq 18 that the system will become a first-order low-pass filter after full compensation for internal and external disturbances.

Design and analysis of repetitive QPR-LADRC control

The control block diagram of the repetitive QPR-LADRC controller is shown in Fig 10. At the front-end, repetitive control and QPR control are parallelly connected to form a repetitive QPR control. The repetitive QPR control can not only eliminate the periodic error contained in the stable closed-loop to improve the steady-state tracking accuracy of the APF system, but also enhance the APF 's ability to suppress specific harmonics. At the back-end, LADRC is connected in series to form repetitive QPR-LADRC composite control. LADRC controller can observe and compensate the current coupling part to achieve high-performance decoupling control, thus, it can further improve the steady-state and dynamic performance of APF.

Fig 10. The structure diagram of repetitive QPR-LADRC control.

As shown in Fig 10, the error e_{l2} is calculated by comparing the reference given value ${i_{l2}}^*$ and the actual output value i_{l2}, and then the system control signal with disturbance is generated by QPR and repetitive controller processing. Finally, the disturbance compensation is carried out by LADRC to form the final system control signal.

Design and analysis of voltage outer loop

Whether the DC-side voltage is stable or not affects the harmonic compensation effect of APF. The PI controller has a simple structure and is easy to control, so it is the most commonly used DC-side voltage control strategy. However, PI control has limited dynamic modulation ability in the face of sudden changes in harmonic load [44]. The quasi-proportional resonant control can enhance the dynamic modulation ability of the system and improve the control effect of the voltage outer loop. Therefore, this paper uses QPR controller to control the voltage outer loop. The specific control block diagram is shown in Fig 11.

Fig 11. Control strategy of the Voltage outer loop.

In Fig 11, the DC-side voltage error e_{dc} is derived by comparing the DC-side voltage reference value ${V_{dc}}^*$ with the actual DC-side voltage V_{dc}, and then the active power current ${i_{dc}}^*$ required for compensation is generated by processing the error signal through the QPR controller. The active power current ${i_{dc}}^*$ is then superimposed onto

the $d-$axis as the control signal for the current inner loop, and finally the APF is controlled through the current inner loop to achieve stable DC-side voltage.

From Fig 11, the expression of the QPR controller can be obtained as follows:

$$G_{QPR2}(s) = k_{p2} + \frac{2k_{r2}w_{c2}s}{s^2 + 2w_{c2}s + {w_{02}}^2} \tag{19}$$

In Eq 19, k_{p2} is positively correlated with the proportional gain of the system. If the value of k_{p2} is too large, it will cause the system to oscillate. If the value is too small, it will reduce the anti-interference ability of the system. Therefore, this paper selects $k_{p2} = 4$. In order to reduce the harmonic content and steady-state error, $k_{r2} = 100$, $w_{02} = 100\pi$, and the control loop bandwidth angle $w_{c2} = 20$ are selected.

Materials and methods

To verify the effectiveness of the proposed dual closed-loop control strategy, a hardware-in-the-loop simulation test is conducted using the RTDS platform and the simulation results are presented. The picture of the RTDS hardware-in-the-loop simulation platform is shown in Fig 12.

Fig 12. RTDS real-time simulation hardware platform.

Furthermore, Table 1 provides some simulation parameters. In order to highlight the superiority of the dual closed-loop control strategy proposed in this paper, an APF model based on the dual closed-loop PI control is set up with the same simulation parameters.

Table 1. Simulation parameters of the APF system.

Parameters	Numerical Value	Parameters	Numerical Value
Grid voltage	0.48 kV	Inverter side inductance	2 mH
Grid side inductance	0.1 mH	Filter capacitance	11 uF
DC-side capacitance	23.5 mF	DC-side bus voltage	1 kV
Gride frequency	50 Hz	Switching frequency	20 kHz
Msximum compensation current	0.15 kA	b_q	500
w_0	7000	w_c	700

Firstly, the steady-state performance of the system is tested. Therefore, it is necessary to ensure that the nonlinear load remains unchanged. Figs 13(a) and 14(a) is the harmonic current tracking diagram of APF system under two different control strategies. Figs 13(b) and 14(b) show the locally amplified harmonic current tracking diagram. Figs 13(c) and 14(c) is the steady-state phase a grid current diagram. Figs 13(d) and 14(d) is the DC-side voltage fluctuation diagram in steady-state.

Fig 13. Steady-state waveforms with the repetitive QPR-LADRC control and QPR control. (a) Filter tracking current. (b) Filter tracking current after local amplification. (c) Phase a grid current. (d) DC-side voltage.

From Figs 13(a) and 14(a), it is evident that both control strategies have good current tracking ability in steady-state, and as illustrated in Figs 13(b) and 14(b), the APF system with repetitive QPR-LADRC and QPR dual closed-loop control can track harmonic currents more closely than the dual closed-loop PI control. Furthermore,

Fig 14. Steady-state waveforms with dual closed-loop PI control. (a) Filter tracking current. (b) Filter tracking current after local amplification. (c) Phase a grid current. (d) DC-side voltage.

Figs 13(c) and 14(c) indicate that the grid current after APF filtering with repetitive QPR-LADRC and QPR control is smoother. Lastly, in Figs 13(d) and 14(d), both control strategies can control the DC side voltage well, and the fluctuation range of the two is similar.

Secondly, the dynamic performance of the system is analyzed. The APF is designed to start compensation at 0.1 s, and the nonlinear load mutates at 0.4 s (the harmonic current suddenly decreases). Figs 15(a) and 16(a) illustrate the harmonic current tracking of the APF during startup, while Figs 15(b) and 16(b) depict the phase a grid current before and after APF compensation. Figs 15(c) and 16(c) present the response of the APF system to sudden changes in nonlinear load, and the process of grid current change in phase a during the sudden change of the nonlinear load are shown in Figs 15(d) and 16(d).

Fig 15. Dynamic waveforms with the repetitive QPR-LADRC control and QPR control. (a) Filter tracking current at APF startup. (b) Phase a grid current at APF startup. (c) Filter tracking current at sudden load change. (d) Phase a grid current at sudden load change.

Fig 16. Dynamic waveforms with dual closed-loop PI control. (a) Filter tracking current at APF startup. (b) Phase a grid current at APF startup. (c) Filter tracking current at sudden load change. (d) Phase a grid current at sudden load change.

From Figs 15(a) and 16(a), it can be seen that the APF system with repetitive QPR-LADRC and QPR control can instantaneously track harmonic currents at startup, while the traditional double closed-loop PI control needs 0.008 s to complete the tracking of harmonic current. And as shown in Figs 15(b) and 16(b), the traditional double closed-loop PI control has 0.2052 kA grid current overshoot, while the repetitive QPR-LADRC and QPR double closed-loop control does not have grid current overshoot, and its grid current is 0.1986 kA when the APF is started. Then, the analysis of the DC-side voltage is conducted. From Figs 17(a) and 18(a), it can be seen that the APF system with dual closed-loop PI control exhibits obvious overshoot and takes a longer time to reach stability during the startup process, while the APF system with repetitive QPR-LADRC and QPR control can achieve DC voltage control without overshoot and quickly reach the stable-state.

From Figs 15(c) and 16(c), it can be seen that the APF system with repetitive QPR-LADRC and QPR control can regain harmonic current tracking within half a cycle when facing nonlinear load perturbations, and it is 0.008 s faster than the traditional dual closed-loop PI control. In Figs 17(c) and 18(c), it can be seen that both control strategies experience voltage overshoot when facing nonlinear load perturbations, but the voltage overshoot of the APF system with repetitive QPR-LADRC and QPR control is smaller at 4 V, while the voltage overshoot of the APF system with dual closed-loop PI control is 5.6 V. Furthermore, the APF system with repetitive QPR-LADRC and QPR dual closed-loop control can recover stability faster, its only takes 0.09s, while the APF system with dual closed-loop PI control needs 0.113s to recover stability.

In summary, the APF system with repetitive QPR-LADRC and QPR dual closed-loop control exhibits superior performance in both steady-state and dynamic performance.

Fig 17. Dynamic waveforms with the repetitive QPR-LADRC control and QPR control. (a) DC-side voltage at APF startup. (b) DC-side voltage after local amplification. (c) DC-side voltage at sudden load change. (d) DC-side voltage after local amplification.

Fig 18. Dynamic waveforms with dual closed-loop PI control. (a) DC-side voltage at APF startup. (b) DC-side voltage after local amplification. (c) DC-side voltage at sudden load change. (d) DC-side voltage after local amplification.

Finally, the harmonic content of the grid current is analyzed, and the specific analysis results are shown in Table 2. It can be seen from Table 2 that when the grid current is 0.198 kA, the grid current THD is 17.76% with a serious waveform distortion. After filtering, the grid current THD of the APF system with repetitive QPR-LADRC and QPR double closed-loop control is reduced to 1.64%, which is 1.84% lower than the APF system with double closed-loop PI control. When the nonlinear load changes suddenly, the grid current is reduced to 0.13 kA, and the grid current THD is 19.25%. The THD of the grid current is reduced to 2.32% after filtering in the APF system with repetitive QPR-LADRC and QPR control, which is 2% lower than the APF system with double closed-loop PI control. In summary, the repetitive QPR-LADRC and QPR double closed-loop control proposed in this paper can effectively improve the harmonic suppression ability of APF.

Table 2. Comparison of harmonic current THD before and after filtering.

Load current	0.198 kA	0.13 kA
Pre-compensation	17.76%	19.25%
PI control	3.48%	4.32%
Repetitive QPR-LADRC	1.64%	2.32%

Table 3 shows the compensation of the 5th, 7th and 11th harmonic currents in the grid before and after adopting two strategies. From Table 3, it can be seen that the 5th, 7th and 11th harmonics in the grid current are greatly reduced and significantly improved by the repetitive QPR-LADRC and QPR control, which is much better than the double closed-loop PI control.

Table 3. Comparison of the 5th, 7th and 11th harmonic current THD before and after filtering.

Harmonic number	5	7	11	THD
Pre-compensation	15.12%	6.29%	2.46%	17.76%
PI control	2.11%	2.01%	1.33%	3.48%
Repetitive QPR-LADRC	0.52%	0.77%	0.56%	1.64%

Conclusion

In this paper, a mathematical model of neutral-point-clamped APF with LCL filter based on grid side current feedback control is established, and a double closed-loop control strategy based on repetitive QPR-LADRC and QPR control is designed. The repetitive QPR-LADRC control is used for the current inner loop, the front-end repetitive QPR control is used to improve the tracking accuracy of APF, and the back-end LADRC is used for high-performance decoupling control. QPR control is used in the voltage outer loop to keep the DC-side voltage stable. Finally, RTDS online

simulation is carried out. The simulation results show that compared with the traditional dual-loop PI control with feedforward decoupling, the dual-loop control strategy proposed in this paper has better dynamic tracking performance in the face of load changes and higher steady-state accuracy in the face of constant load. It significantly enhances the harmonic suppression capability of the APF system.

Acknowledgments

I sincerely thank my mentor and the members of the research team for their cooperation and assistance. I also want to express my gratitude to Liaoning University of Technology and Liaoning Rongxin Xingye Electric Power Technology Co., Ltd. for their support of this research.

References

1. Zhou X, Cui Y, Ma Y. Fuzzy linear active disturbance rejection control of injection hybrid active power filter for medium and high voltage distribution network. IEEE Access. 2021;9:8421–8432.

2. Alasali F, Nusair K, Foudeh H, Holderbaum W, Aziz A. Modern optimal controllers for hybrid active power filter to minimize harmonic distortion. Electronics. 2022;11(9):1453.

3. Das SR, Ray PK, Sahoo AK, Ramasubbareddy S, Babu TK, Kumar NM, Elavarasan RM, Mihet-Popa L. A comprehensive survey on different control strategies and applications of active power filters for power quality improvement. Energies. 2021;14(15):4589.

4. Li H, Qu Y, Lu J, Li S. A composite strategy for harmonic compensation in standalone inverter based on linear active disturbance rejection control. Energies. 2019;12(13):2618.

5. Kashif M, Hossain MJ, Zhuo F, Gautam S. Design and implementation of a three-level active power filter for harmonic and reactive power compensation. Electr. Power Syst. Res. 2018;165:144–156.

6. Ghoudelbourk S, Azar AT, Dib D. Three-level (NPC) shunt active power filter based on fuzzy logic and fractional-order PI controller. Int. J. Autom. Control. 2021;15(2):149–169.

7. Mhawi E, Daniyal H, Sulaiman MH. Advanced techniques in harmonic suppression via active power filter: A review. Int. J. Power Electron. Drive Syst. 2015;6(2):185–195.

8. Hoon Y, Mohd Radzi MA, Hassan MK, Mailah NF. Control algorithms of shunt active power filter for harmonics mitigation: A review. Energies. 2017;10(12):2038.

9. Zhang M, Wen JM, Wu SY, Guan YC, Feng CS, Lu JH. Dual-loop control of hybrid active power filter. Electr. Mach. Control. 2023;27:119–126.

10. Zeng Z, Yang J, Yu N. Research on PI and repetitive control strategy for Shunt Active Power Filter with LCL-filter. In: Proceedings of The 7th International Power Electronics and Motion Control Conference. vol. 4. IEEE; 2012.p. 2833–2837.

11. Li S, Deng N, Zheng J, Yu J. Control Strategy of Photovoltaic Grid Connected System Based on PI and QPR Double Closed Loop Control. In: Journal of Physics: Conference Series. vol. 2136. IOP Publishing; 2021.p. 012017.

12. Sou WK, Chao CW, Gong C, Lam CS, Wong CK. Analysis, design, and implementation of multi-quasi-proportional-resonant controller for thyristor-controlled LC-coupling hybrid active power filter (TCLC-HAPF). IEEE Trans. Ind. Electron. 2021;69(1):29–40.

13. Yan X, Shao W, Cheng Y, Li N. Study of Active Power Filter Control Strategy based on Improved PQ Harmonic Current Detection. In: 2020 Asia Energy and Electrical Engineering Symposium (AEEES). IEEE; 2020.p. 280–284.

14. Li S, Zhou S, Li H. Harmonic Suppression Strategy of LCL Grid-Connected PV Inverter Based on Adaptive QPR_PC Control. Electronics. 2023;12(10):2282.

15. Gao Y, Zhu L, Wang X, Lv X, Wei H. A Novel Repeat PI Decoupling Control Strategy with Linear Active Disturbance Rejection for a Three-Level Neutral-Point-Clamped Active Power Filter with an LCL Filter. Electronics. 2024;13(15):2973.

16. Zhou S, Liu J, Zhou L, Zhang Y. DQ current control of voltage source converters with a decoupling method based on preprocessed reference current feed-forward. IEEE Trans. Power Electron. 2017;32(11):8904-8921.

17. Li X, Li B. A decoupling control method for shunt hybrid active power filter based on generalized inverse system. J. Electr. Comput. Eng. 2017;2017(1):5232507.

18. Liu Y, Zeng L, Pan H, Yang T. Research of [A]ctive Power Filter Using Improved Backstepping Active disturbance rejection control strategy. In: 2021 China Automation Congress (CAC). IEEE; 2020.p. 8001–8006.

19. Han J. From PID to active disturbance rejection control. IEEE Trans. Ind. Electron. 2009;56(3):900–906.

20. Gao Z. Scaling and bandwidth-parameterization based controller tuning. In: Proceedings of the American Control Conference (ACC). vol. 6. 2003.p. 4989–4996.

21. Qin D, Zeng G. Research on parallel shunt hybrid active power filter with repetitive LADRC algorithm. In: 2015 IEEE 2nd International Future Energy Electronics Conference (IFEEC). IEEE; 2015.p. 1–6.

22. Wu H, Wang Y, Liu B, Chen L, Pei G. Research and improvement of current control strategy of active power filter. In: IOP Conference Series: Earth and Environmental Science. vol. 619. IOP Publishing; 2020.p. 012069.

23. Zhang H, Xian J, Shi J, Wu S, Ma Z. High performance decoupling current control by linear extended state observer for three-phase grid-connected inverter with an LCL filter. IEEE Access. 2020;8:13119–13127.

24. Bosch S, Staiger J, Steinhart H. Predictive current control for an active power filter with LCL-filter. IEEE Trans. Ind. Electron. 2017;65(6):4943–4952.

25. Xu AJ, Xie BS, Kan CJ, Ji DL. An improved inverter-side current feedback control for grid-connected inverters with LCL filters. In: 2015 9th International Conference on Power Electronics and ECCE Asia (ICPE-ECCE Asia). IEEE; 2015.p. 984–989.

26. Pal B, Sahu PK, Mohapatra S. A review on feedback current control techniques of grid-connected PV inverter system with LCL filter. In: 2018 Technologies for Smart-City Energy Security and Power (ICSESP). 2018.p. 1–6.

27. Zhou X, Xu H, Ma Y, Hu Y. Research on Control Strategy Based on Second-order Linear Active Disturbance Rejection Control for LCL-type Grid-connected Inverter. In: 2022 IEEE International Conference on Mechatronics and Automation (ICMA). IEEE; 2022.p. 864–869.

28. Benrabah A, Xu D, Gao Z. Active disturbance rejection control of LCL-filtered grid-connected inverter using Padé approximation. IEEE Trans. Ind. Appl. 2018;54(6):6179–6189.

29. Xuesong Z, Yulong C, Youjie MA, Yangyang C, Xiaotong S, Jianpeng G. Improved Active Disturbance Rejection Control of Wind Power Grid-connected Inverter Based on Nonlinear ESO. Electric Drive. 2022;52(5):052.

30. Feng Z, Zhou X, Wang C, Huang W, Chen Z. LADRC-based grid-connected control strategy for single-phase LCL-type inverters. Plos one. 2024;19(5):e0303591.

31. Liserre M, Blaabjerg F, Hansen S. Design and control of an LCL-filter-based three-phase active rectifier. IEEE Trans. Ind. Appl. 2005;41(5):1281–1291.

32. Ye T, Dai NY, Lam CS, Wong MC, Guerrero JM. Analysis, design, and implementation of a quasi-proportional-resonant controller for a multifunctional capacitive-coupling grid-connected inverter. IEEE Trans. Ind. Appl. 2016;52(5):4269–4280.

33. Wu Z, Xu G, Zhu W, Sheng G. Study on Dispersing BPF-QPR Harmnoic Current Control for Active Power Filter. In: 2023 IEEE PELS Students and Young Professionals Symposium (SYPS). IEEE; 2023.p. 1–5.

34. Li Y, Zhang J, Hao Z, Tian P. Improved pr control strategy for an lcl three-phase grid-connected inverter based on active damping. Applied Sciences. 2021;11(7):3170.

35. Liu Z, Huang W, Liu S, Yang Y. An Improved Hydraulic Energy Storage Wave Power-Generation System Based on QPR Control. Energies. 2023;16(2):647.

36. Wu Z, Xu G, Zhu W, Sheng G. An improved proportional resonant control strategy in the static coordinate for four-leg active power filters. In: Zhongguo Dianji Gongcheng Xuebao(Proceedings of the Chinese Society of Electrical Engineering). vol. 32. Chinese Society for Electrical Engineering; 2012.p. 113–120.

37. Tang Y, Loh PC, Wang P, Choo FH, Gao F, Blaabjerg F. Generalized design of high performance shunt active power filter with output LCL filter. IEEE Tran. Ind. Electron. 2011;59(3):1443–1452.

38. Garcia-Cerrada A, Pinzon-Ardila O, Feliu-Batlle V, Roncero-Sanchez P, Garcia-Gonzalez P. Application of a repetitive controller for a three-phase active power filter. IEEE Trans. Power Electron. 2007;22(1):237–246.

39. Mattavelli P, Marafao FP. Repetitive-based control for selective harmonic compensation in active power filters. IEEE Tran. Ind. Electron. 2004;51(5):1018–1024.

40. Rueffer BS. Small-gain conditions and the comparison principle. IEEE Tran. Autom. Control. 2010;55(7):1732–1736.

41. Zhou X, Wang J, Ma Y. Linear active disturbance rejection control of grid-connected photovoltaic inverter based on deviation control principle. Energies. 2020;13(15):3790.

42. Yin Y. Immunity of shunt active power filter based on improved LADRC. Trans. Inst. Meas. Control. 2024;46(2):326–333.

43. He G, Chen G, Dong Y, Li G, Zhang W. Research on LADRC of Grid-Connected Inverter Based on LCCL. Energies. 2022;15(13):4686.

44. He Y, Liu J, Tang J, Wang Z, Zou Y. Research on control system of DC voltage for active power filters with three-level NPC inverter. In: 2008 Twenty-Third Annual IEEE Applied Power Electronics Conference and Exposition. IEEE; 2008.p. 1173–1178.
